# Association of gadolinium-enhanced magnetic resonance imaging with hepatic fibrosis and inflammation in primary sclerosing cholangitis

**DOI:** 10.1371/journal.pone.0193929

**Published:** 2018-03-07

**Authors:** Sarah Keller, Annette Aigner, Roman Zenouzi, Anne C. Kim, Arnoud Meijer, Sören A. Weidemann, Till Krech, Ansgar W. Lohse, Gerhard Adam, Christoph Schramm, Jin Yamamura

**Affiliations:** 1 Department of Diagnostic and Interventional Radiology and Nuclear Medicine, University Medical Center Hamburg-Eppendorf (UKE), Hamburg, Germany; 2 Institute of Medical Biometry and Epidemiology, University Medical Center Hamburg-Eppendorf (UKE), Hamburg, Germany; 3 1st Department of Medicine, University Medical Center Hamburg-Eppendorf (UKE), Hamburg, Germany; 4 Department Stroke and Neurovascular Imaging, The Permanente Medical Group, San Francisco, California, United States of America; 5 Department of Radiology, Leiden University Medical Center (LUMC), Leiden, The Netherlands; 6 Institute of Pathology, University Medical Center Hamburg-Eppendorf (UKE), Hamburg, Germany; University of Chicago, UNITED STATES

## Abstract

**Objective:**

To evaluate magnetic resonance imaging (MRI) parameters T2 signal, contrast enhancement (CE), and relative liver enhancement (RLE) of extracellular gadolinium-based contrast agent (GBCA)-enhanced MRI as a marker for hepatic fibrosis and inflammation in patients with primary sclerosing cholangitis (PSC).

**Methods:**

3.0-Tesla MRI scans and liver biopsies of 40 patients (41.2 ± 17.1 years) were retrospectively reviewed. Biopsies were obtained within a mean time of 54 ± 55 days to MRI scans and specimens were categorized according to Ishak modified hepatic activity index (mHAI) and Scheuer staging of fibrosis. T2 signal (N = 40), CE alterations (N = 29), and RLE (N = 29) were assessed by two raters. Mixed-effects regression models were applied to estimate the association between histopathology and MRI parameters.

**Results:**

No significant association was observed between T2 signal or CE alterations with stages of fibrosis or mHAI grading. Regression models revealed significant positive associations of portal venous phase RLE with mHAI grade ≥ 7 points [β = 25.5; 95% CI (2.53; 48.62); p = 0.04] and delayed phase RLE with stages of fibrosis [stage 2: β = 35.13; 95% CI (11.35; 58.87); p = 0.007; stage 3/4: β = 69.24; 95% CI (45.77; 92.75); p < 0.001]. The optimal cut-off value of 66.6% delayed phase RLE distinguished fibrosis stages 0–2 from 3–4 with a sensitivity of 0.833 and specificity of 0.972. Inter-rater reliability (IRR) for quantification of RLE was ‘excellent’ (r = 0.90–0.98). IRR was ‘substantial’ for detection of T2 signal in the right liver lobe (RL) (Kappa = 0.77) and ‘almost perfect’ for T2 signal of the left liver lobe (LL) and CE of both lobes (Kappa = 0.87–1.0).

**Conclusion:**

The simple and reproducible method of RLE quantification on standard extracellular GBCA-enhanced MRI may provide a correlate measure of advanced stages of hepatic fibrosis and potentially also inflammation in PSC patients, if validated in larger cohorts.

## Introduction

Primary sclerosing cholangitis (PSC) is a cholestatic liver disease characterized by chronic inflammation, subsequent obliterative periductal destruction and fibrosis, culminating in secondary biliary cirrhosis and liver failure [1–3].

Quality of life and patient survival are reduced due to a markedly increased risk of cholangiocarcinoma, hepatocellular carcinoma, and colorectal cancer [4, 5]. The incidence of PSC varies geographically, where the reported incidence in the United States, Northern Europe, and Canada ranges from 0.9 to 1.3 per 100,000, and is less than 0.1 per 100,000 in Southern Europe and Asia [6, 7].

The diagnosis of PSC is usually based on biochemical, clinical, and cholangiographic findings using magnetic resonance cholangiopancreaticography (MRCP) [8–10]. Histopathological examination is not standard procedure in current guidelines [9]. However, histopathological scoring systems for inflammatory activity and fibrosis can provide useful information about disease activity and stage of liver fibrosis as well as the patient´s prognosis [11].

PSC is unique in its patchy distribution of liver parenchyma inflammation and confluent fibrosis, increasing the risk of sampling error, as biopsy covers only a tiny fraction of the liver (roughly 1/50,000^th^) [12]. Furthermore, its invasive nature carries risks such as hemorrhage with a fatality rate of up to 0.03% [13]. Non-invasive tools to quantify fibrosis and liver inflammation are urgently needed to assess treatment response in clinical trials.

Magnetic resonance imaging (MRI) is recommended as the diagnostic modality of choice in PSC [14]. In recent years elaborate MRI sequences for diagnosis and staging of fibrosis, such as MR elastography (MRE) have been investigated. MRE non-invasively quantifies the stiffness of liver in later stages of fibrosis (2 vs. 3 vs. 4) but may be confounded by a variety of factors, which can alter liver stiffness such as hepatic steatosis, cholestasis, and portal hypertension [15–17].

Conventional extracellular gadolinium-based contrast agent (GBCA)-enhanced MRI and MRCP are routinely performed in PSC patients for diagnosis and surveillance of malignancy and dominant biliary strictures [18–20]. An additional imaging feature of GBCA-enhanced MRI is the evaluation and quantification of inflammation and progressive fibrosis using either hepatocyte-specific or extracellular GBCA [21, 22]. Abnormal perfusion patterns using extracellular GBCA have been observed in patients with hepatic inflammation in acute [23, 24] and chronic liver diseases [25, 26] with distinct patterns of fibrosis, inflammatory cell infiltrates, necrosis, and micro- or macronodular cirrhosis. A multiphase extracellular GBCA-enhanced MRI study performed by Martin et al. [27] in patients with hepatitis reported a high correlation of delayed phase contrast enhancement with histological staging of fibrosis (r = 0.96; 95% confidence interval (CI) 0.941–0.976), but not between arterial/venous/delayed phase imaging and histological grades of inflammation. In recent years dynamic contrast-enhanced (DCE) MRI using the hepatocyte-specific contrast agent gadoxetic acid has been proposed to assess hepatic function as a surrogate for hepatic fibrosis, by quantifying parameters like hepatocellular uptake rate, input relative blood flow, and mean transit time (MTT) using deconvolutional analyses [28]. For example Nilsson et al. [28] described a significant lower global median hepatic extraction fraction (HEF) and global median MTT, and larger parenchymal volume (p< 0.05) in patients with liver cirrhosis compared to healthy controls. Norén et al. [29] reported significant intergroup differences in hepatic contrast uptake rate (K(hep)) between mild (F0/1) and advanced (F3/4) histopathological stages of fibrosis (p = 0.05) in N = 38 patients with various liver diseases. However the routine clinical application of functional DCE imaging is limited due to extensive and time-consuming post-processing, as well as to several confounding factors such as cardiac status, hepatic congestion, and inflammation [30].

The aim of the present study was to retrospectively evaluate the quantitative and qualitative MRI parameters T2 signal, contrast enhancement (CE) and relative liver enhancement (RLE) of extracellular GBCA-enhanced MRI as a marker for hepatic fibrosis and inflammation in patients with PSC using liver histopathology as gold standard.

## Methods

### Subjects

The retrospective study was approved by the Ethics committee of the Hamburg Medical Association. 40 patients with known PSC according to EASL guidelines were assessed between January 2008 and January 2015. Written informed consent for general use of data and standard MRI was acquired from each patient. Patients with previous liver surgery, a history of hepatocellular carcinoma or cholangiocarcinoma, and a change of the therapeutic regimen for PSC disease within the time interval of MRI scan and liver biopsy, were excluded. All patients underwent liver biopsy of the right liver lobe (RL) and/or the left liver lobe (LL) within 6 months (mean ± standard deviation 54 ± 55 days, range 0–180 days) to the MRI scan.

Out of 40 patients, N = 29 patients received intravenous contrast agents. The administration of contrast agents was avoided in N = 8 patients due to renal impairment (glomerular filtration rate < 30 ml/min) and N = 1 patient due to relevant allergic reactions after previous contrast administration. Two patients received a hepatocyte-specific contrast agent and were excluded from contrast-based analysis (CE and RLE).

### Liver biopsy and histopathological examination

Because of previous reports of higher sensitivity for detection of fibrosis and lower complication rate with mini-laparoscopic versus percutaneous performed liver biopsies [31], all samples were acquired by mini-laparoscopy as previously described [32]. The location of biopsy was documented in the laparoscopy report and retrospectively allocated according to liver segments defined by Strasberg et al. [33]. The histopathology was evaluated by two pathologists with 5 years (SW) and 11 years (TK) experience in histopathological grading/staging of liver biopsies separately for the RL and LL, using the Ishak modified hepatic activity index (mHAI) and Scheuer fibrosis staging of chronic hepatitis [34]. The mHAI was chosen since there is no grading system specific for PSC inflammatory activity. The mHAI grades four major characteristics (sum 18 points) of necroinflammatory tissue changes (A: periportal or periseptal interface hepatitis; B: confluent necrosis; C: focal lytic necrosis, apoptosis and focal inflammation; D: portal inflammation). In autoimmune hepatitis, a cut-off value of mHAI points ≥ 3 represents significant liver inflammation [35], and was found to indicate immunosuppressive treatment in patients with PSC in a previous retrospective multicenter study [36]. The staging system developed by Scheuer et al. [37] was used for architectural staging of liver fibrosis and cirrhosis. This classification system defines liver tissue without architectural changes (0 points) to cirrhosis (4 points) [37].

### MR imaging technique

MRI was performed on a 3.0-Tesla scanner (Ingenia, Philips Medical Systems, Best, The Netherlands) with an eight-channel body coil. Axial T2-weighted imaging was performed covering the whole liver and subjects were asked to hold their breath at the same depth during each acquisition. Baseline and contrast-enhanced dynamic images were acquired before and after intravenous injection of 0.1 mmol/kg (0.2 ml/kg) of gadopentetate dimeglumine (Magnevist Bayer Healthcare, Leverkusen, Germany) into the antecubital vein using a power injector (Medrad, Spectris Solaris, EP Injection System, Bayer Healthcare, Berlin, Germany) at an infusion rate of 2 mL/s, followed immediately by a bolus of 20 mL saline (NaCl 0.9%) at the same infusion rate. A fat saturated T1-weighted imaging of the whole liver in axial orientation was performed before contrast injection and followed by repetitive sequences 10 seconds (arterial phase), 90 seconds (portal venous phase) and 5 minutes (delayed phase) after injection. MRI sequence details are specified in Table 1.

**Table 1:** Sequence parameters for MRCP, T1- and T2-weighted MR imaging.

Sequence	Plane	Field of view (mm)	Flip angle (°)	TE (ms)	TR (ms)
T1w (fat saturated)	axial	340x340	10	1.6	3.3
T2w	axial	340x340	90	80	1250
T2w	coronal	340x340	90	80	875

Contrast agent: Gadopentetate dimeglumine i.v. 0.1 mmol/kg (0.2 ml/kg).

Image acquisition before (non-enhanced), 10 seconds (arterial), 90 seconds (portal venous), and 5 minutes (delayed) after injection.

### Image analysis

MRI data were analyzed and rated by two independent radiologists (SK and JY) with 4 and 10 years experience in abdominal MRI, respectively, blinded to patients’ history, histology, and laboratory findings. Images with binary results between both raters were assessed by a third rater (AM) with 6 years experience in abdominal imaging.

**Fig 1:**
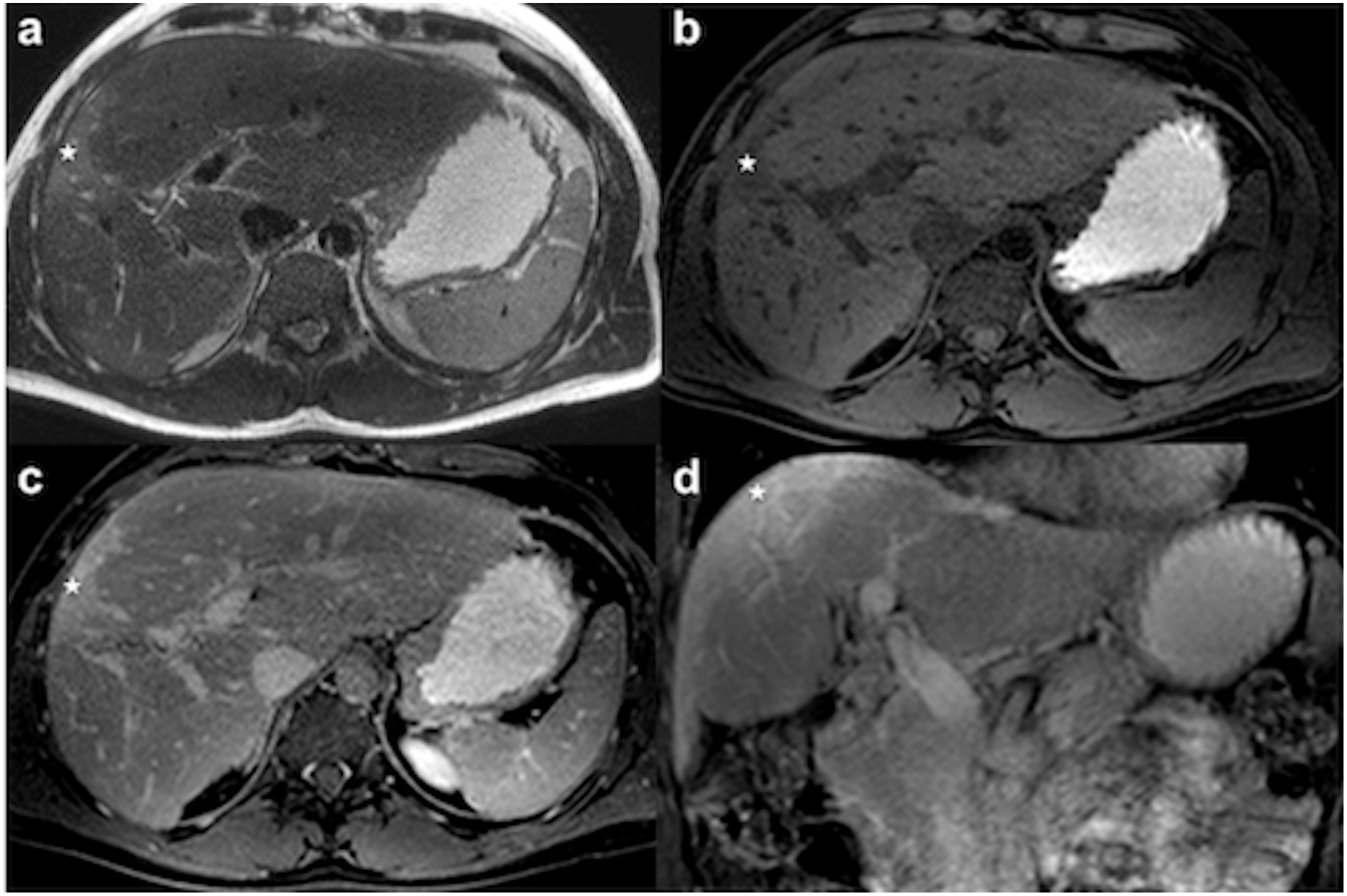
Wedge-shaped sub capsular area (asterisk) of T2 hyperintensity and GBCA accumulation on delayed phase imaging of a 39-year old male patient. (a) Axial T2-weighted image; (b) Non-enhanced axial T1-weighted fat suppressed image; (c, d) T1-weighted delayed phase axial and coronal image.

According to the liver segment used for laparoscopically performed biopsy, axial and coronal T2-weighted images and contrast-enhanced axial T1-weighted images were analyzed regarding the visual aspect of T2 signal alterations (T2 hyperintensity = 1; absence of significant T2 hyperintensity = 0) and increased contrast enhancement (CE) (increased CE = 1; absence of significant CE = 0) of the portal venous and delayed phase imaging (Fig 1).

**Fig 2:**
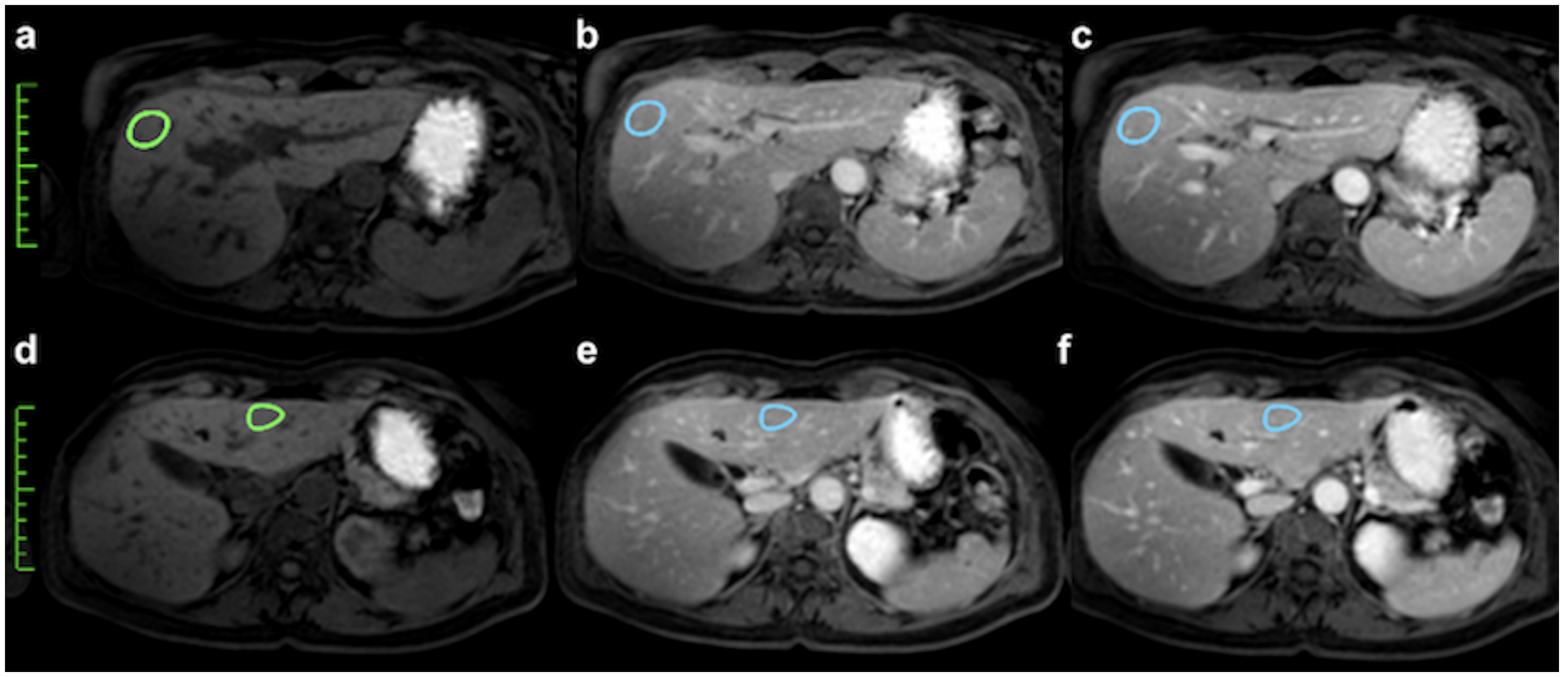
Bolus-timed contrast-enhanced axial T1-weighted breath-hold imaging of a 37-year old female patient. (a) Determination of the relative liver enhancement (RLE) using manually localized regions of interest (ROI) in liver segments of the right lobe (upper row) and left lobe (lower row) according to biopsy location. T1-weighted non-enhanced axial image (a, d); T1-weighted portal venous phase axial image (b, e); T1-weighted delayed phase axial image (c, f).

To avoid sampling errors, three regions of interest (ROI), with a mean size of 1.8 cm^2^ (± 0.7 cm^2^ standard deviation) were drawn on adjacent slices of the non-enhanced T1-weighted axial imaging of the biopsied liver lobe. Each ROI was then copied exactly to the portal venous and delayed phase axial contrast-enhanced T1-weighted image. The signal intensity (SI) of the copied ROIs was assessed to derive three replicate values, which were then averaged for further assessment (Fig 2).

Mean SI obtained from the ROIs of the T1-weighted non-enhanced (baseline) and contrast-enhanced (portal venous and delayed phase) images were used to calculate the RLE using the following algorithm, adapted for extracellular GBCA and published previously by Nolz et al. [38]:

$$RLE=\frac{\left({SI}_{enhanced}-{SI}_{non-enhanced\;}\right)}{{SI}_{non-enhanced}}\;x\;100$$

Where *SI*_*enhanced*_ is the signal intensity either of the portal venous or the delayed phase contrast-enhanced imaging, and *SI*_*non-enhanced*_ is the signal intensity of the baseline image.

### Laboratory data

Relevant demographic and clinical data, as well as serum liver function tests of disease activity (alkaline phosphatase (AP), alanine-amino-transferase (ALT), aspartate-amino-transferase (AST), Immunoglobulin G (IgG), gamma-glutamyltransferase (γGT)) were obtained from the patients’ records at the time point closest to MRI examination (mean 25.6 ± 27.6 days, range 0–125 days).

### Statistical analysis

To quantify the inter-rater reliability (IRR) of the two observers we used Cohen’s Kappa with its 95% confidence interval (CI) [39]. The Kappa coefficient amplitude was 0 to 1, where the agreement strength was interpreted using guidelines by Landis and Koch [40]: <0, poor; 0.01 to 0.20, slight; 0.21 to 0.4, fair; 0.41 to 0.6, moderate; 0.61 to 0.80, substantial; 0.81 to 1, almost perfect. For binary results of T2 signal and CE rating, the results of an independent third rater were used.

For continuous variables (RLE portal venous and delayed) Pearson correlation coefficients (r) and Bland-Altman plots were used to assess the IRR of the two observers. The criteria of Portney and Watkins [41] were used to judge the strength of the correlation coefficients, as follows: little to no relationship (r ≤ 0.25), fair degree of relationship (r = 0.26 to 0.50), moderate-to-good relationship (r = 0.51 to 0.75), and good-to-excellent relationship (r ≥ 0.76).

The dependencies in the data for measurements in the RL and LL were quantified descriptively for mHAI and fibrosis stage. Due to these dependencies we used mixed-effects logistic and linear regression models with a random intercept by patient to derive effect estimates and 95% confidence intervals (CI) for the association between CE, T2, RLE portal venous and RLE delayed as dependent variables, and mHAI and fibrosis as independent variables. For sensitivity analyses we also performed these analyses also with laboratory parameters as independent variables, and derived an optimal cut-off value with highest sensitivity and specificity for the distinction between fibrosis stages 0/1 to 2 and 3 to 4 based on RLE delayed.

For all analyses, the statistical software R [42] was used, Cohen`s Kappa was based on the implementation in the irr [43] package, mixed-effects models on the lme4 [44] and the lmerTest package [45], optimal cut-off plots and derivation on ROCR [46], and pROC [47] packages.

## Results

### Patient cohort and laboratory results

Out of 40 patients included in this study, N = 29 patients received a contrast-enhanced MRI scan. Their data were analyzed both for signal increase on T2-weighted images, CE alterations and RLE. The remaining N = 11 non-contrast-enhanced MRI scans were analyzed for signal alterations only on T2-weighted imaging. Patient characteristics, histopathological and MRI parameters are included in Table 2.

**Table 2:** Demographics, histopathological findings and MRI parameters [contrast enhancement (CE), T2 hyperintensity (T2) and relative liver enhancement (RLE)] obtained in biopsied segments of the right (RL) and left (LL) liver lobe.

		**Mean ± SD**		**Total count (N)**
**Age (years)**		41.2 ± 17.1		40
**Gender male: female**		23:17		
**Histology** *[RL and LL*: *N = 26; RL*: *N = 8; or LL*: *N = 6****]***		Count N (%*)		
**mHAI**	**Grade/Stage**	**Right lobe**	**Left lobe**	**Count (N)**
	0–2	19 (55.88%)	19 (59.38%)	RL: 34, LL: 32
	3–6	10 (29.41%)	10 (31.25%)	
	≥7	5 (14.71%)	3 (9.38%)	
**Fibrosis**	0	6 (17.65%)	6 (18.75%)	RL: 34, LL: 32
	1	13 (38.24%)	12 (37.5%)	
	2	6 (17.65%)	8 (25.0%)	
	3	3 (8.82%)	4 (12.5%)	
	4	6 (17.65%)	2 (6.25%)	
**MR imaging parameters**	**No/yes**			
**CE** *[RL and LL*: *N = 19; RL*: *N = 6; LL*: *N = 4]*	**0**	11 (44.0%)	11(47.83%)	RL: 25, LL: 23
	**1**	14 (56.0%)	12 (52.17%)	
**T2** *[RL and LL*: *N = 26; RL*: *N = 8; LL*: *N = 6]*	**0**	15 (44.12%)	18 (56.25%)	RL: 34, LL: 32
	**1**	19 (55.88%)	14 (43.75%)	
**Relative liver enhancement** *[RL and LL*: *N = 19; RL*: *N = 6; LL*: *N = 4]*		**Mean +/- SD**		
**RLE** (portal venous) (%)		69.1 (25.3)	62.7 (24.8)	RL: 25, LL: 23
**RLE** (delayed) (%)		34.0 (36.8)	33.1 (37.6)	RL: 25, LL: 23

*Percentages might not add up 100% as they are rounded to the nearest percent.

Abbreviations: mHAI, modified hepatic activity index; RLE; relative liver enhancement (%); RL, right liver lobe; LL, left liver lobe; CE, contrast enhancement; T2, signal on T2-weighted axial image.

Nominal scoring: 0 = no CE alterations/T2 hyperintensity; 1 = CE alterations/T2 hyperintensity.

### Liver histology

Histopathological results were available from all patients and obtained either from the RL (N = 8) or LL (N = 6) or both liver lobes (N = 26). In total, 66 biopsies were analyzed (RL: 34; LL: 32) (Table 2).

According to histopathological staging of liver fibrosis, 8/66 biopsies obtained from the LL or RL were classified as liver cirrhosis (stage 4), 21/66 biopsies showed a significant to severe stage of fibrosis (stage 2 or 3) and 37/66 no or minor fibrotic tissue alterations (stage 0 or 1) (Table 2).

The highest mHAI of this patient cohort was 9 of 18 points and found in 1 biopsy. 28 biopsies (RL N = 15; LL N = 13) were classified having an elevated mHAI, based on the cut-off value ≥ 3.

We found interdependencies in the data for measurements in the RL and LL for both fibrosis stage and mHAI, quantified with a Spearman correlation coefficient between LL and RL of r = 0.70 for fibrosis and r = 0.71 for mHAI (Data are illustrated in S1 Table).

### Relative liver enhancement in portal venous and delayed phase images

The mean ± standard deviation (SD) of RLE in the portal venous phase was 69.1 ± 25.3% (RL) and 62.7 ± 24.8% (LL), and 34.0 ± 36.8% (RL) and 33.1 ± 37.6% (LL) in the delayed phase imaging, respectively (Table 2).

### Association between relative liver enhancement and histological stages of fibrosis

**Fig 3:**
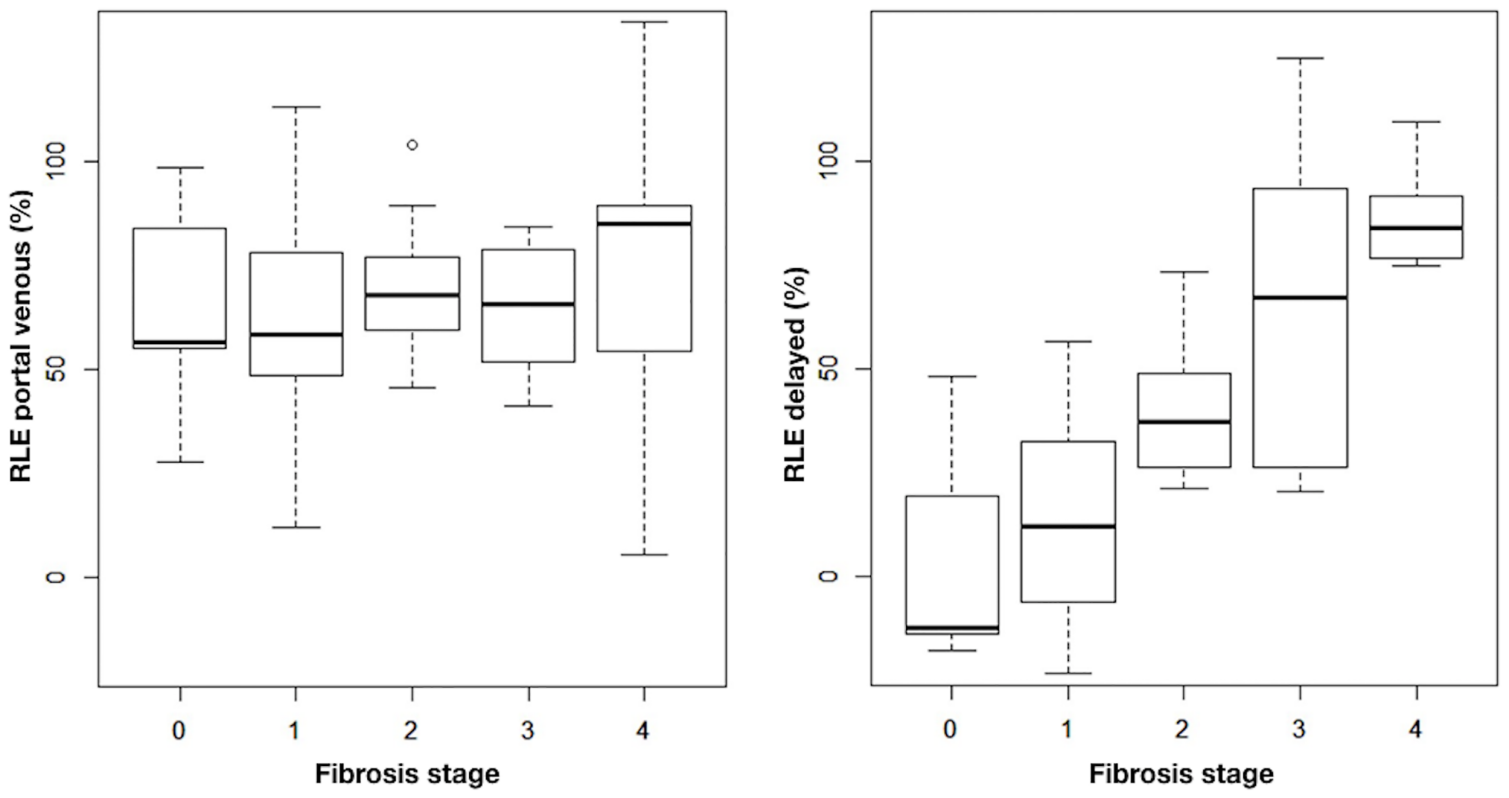
Boxplot analysis of the portal venous phase (left) and delayed phase (right) relative liver enhancement (RLE) (y-axis) according to fibrosis stages (0–4, x-axis), averaged for the right (RL) and left (LL) liver lobe.

The associations between RLE of the portal venous and delayed phase imaging and fibrosis stages are depicted in Fig 3. Using regression analysis, no significant associations of the portal venous phase RLE and stages of fibrosis were observed (p ≥ 0.72) (Table 3).

**Table 3:** Association between imaging parameters (CE, T2, RLE portal venous, RLE delayed) and histological inflammation and fibrosis based on mixed-effects models.

	Contrast-enhancement^1^(N = 48)	T2 hyperintensity^1^ (N = 66)	RLE pvp^2^ (N = 48)	RLE dp^2^ (N = 48)
	OR (p-value)		Regression coefficient β (p-value)	
**Inflammation**^3^				
mHAI 0–2	0.24 (0.10)	0.38 (0.40)	4.16 (0.59)	-8.54 (0.40)
mHAI 3–6	0.22 (0.11)	0.32 (0.33)	-2.60 (0.74)	-13.22 (0.22)
mHAI ≥7	0.28 (0.33)	0.84 (0.93)	**25.5 (0.04)**	14.64 (0.45)
**Fibrosis**^4^				
Stage 1	1.12 (0.92)	1.74 (0.76)	-4.20 (0.72)	9.19 (0.39)
Stage 2	1.62 (0.71)	4.34 (0.46)	4.27 (0.75)	**35.13 (0.007)**
Stage 3 and 4	8.79 (0.12)	532.46 (0.13)	-0.23 (0.99)	**69.24 (<0.001)**

Abbreviations: mHAI, modified hepatic activity index; T2, T2-weighted image; CE, contrast-enhancement; RLE, relative liver enhancement, OR, Odds ratio; β, regression coefficient; pvp, portal venous phase; dp, delayed-phase.

^1^ based on mixed-effect logistic regression models with patient as random intercept, imaging (CE, T2) as dependent variables, each histology parameter as independent variable.

^2^ based on mixed-effects linear regression models with patient as random intercept, RLE (portal venous phase, delayed phase) as dependent variables.

^3^ Reference is mHAI grade 0–2

^4^ Reference is fibrosis stage 0.

**Bold font**: significant p-value ≤ 0.05.

However, there was a consistent positive association between the delayed phase RLE and fibrosis stage. This association was also found significant based on regression analysis [fibrosis stage 2: β = 35.13; 95%-CI (11.35; 58.87); p = 0.007); fibrosis stage 3/4: β = 69.24; 95% CI (45.77; 92.75); p < 0.001)] (Table 3).

### Sensitivity analyses

**Fig 4:**
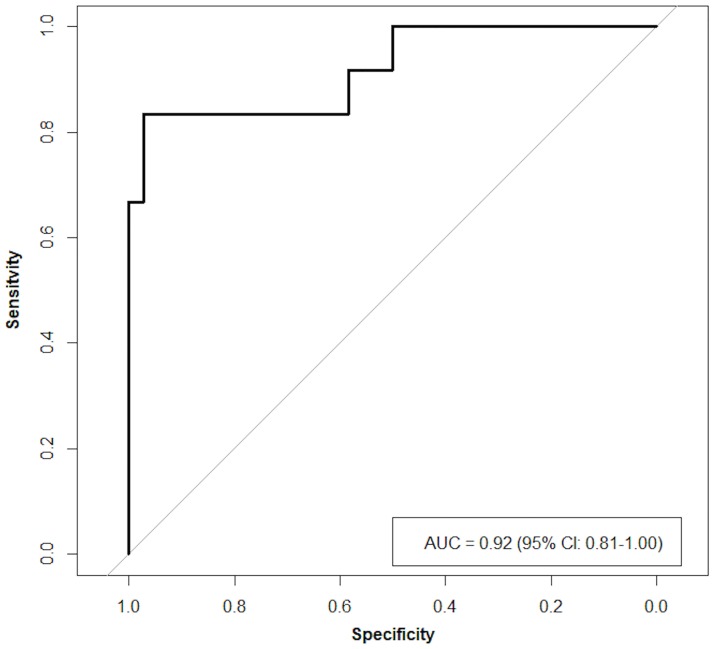
Receiver operating characteristic (ROC)-curve of the RLE delayed derived an optimal cut-off value of 66.6% with a sensitivity of 0.833 and a specificity of 0.972 (area under the curve (AUC) 0.92) for distinguishing between fibrosis grade 0–2 and fibrosis grade 3–4.

Based on the significant results for RLE delayed, we derived an optimal cut-off value of 66.6%, with a sensitivity of 0.833 and a specificity of 0.972 for distinguishing between fibrosis grade 0–2 and fibrosis grade 3–4. A higher sensitivity of 0.917 can only be obtained by compromising specificity to 0.583 based on a cut-off of 26.43% for the RLE delayed (Fig 4). Overall, an area under the curve (AUC) value of 0.92 showed that the RLE delayed performed well in distinguishing between these fibrosis stage groups.

### Relative liver enhancement and histopathological grade of inflammation

**Fig 5:**
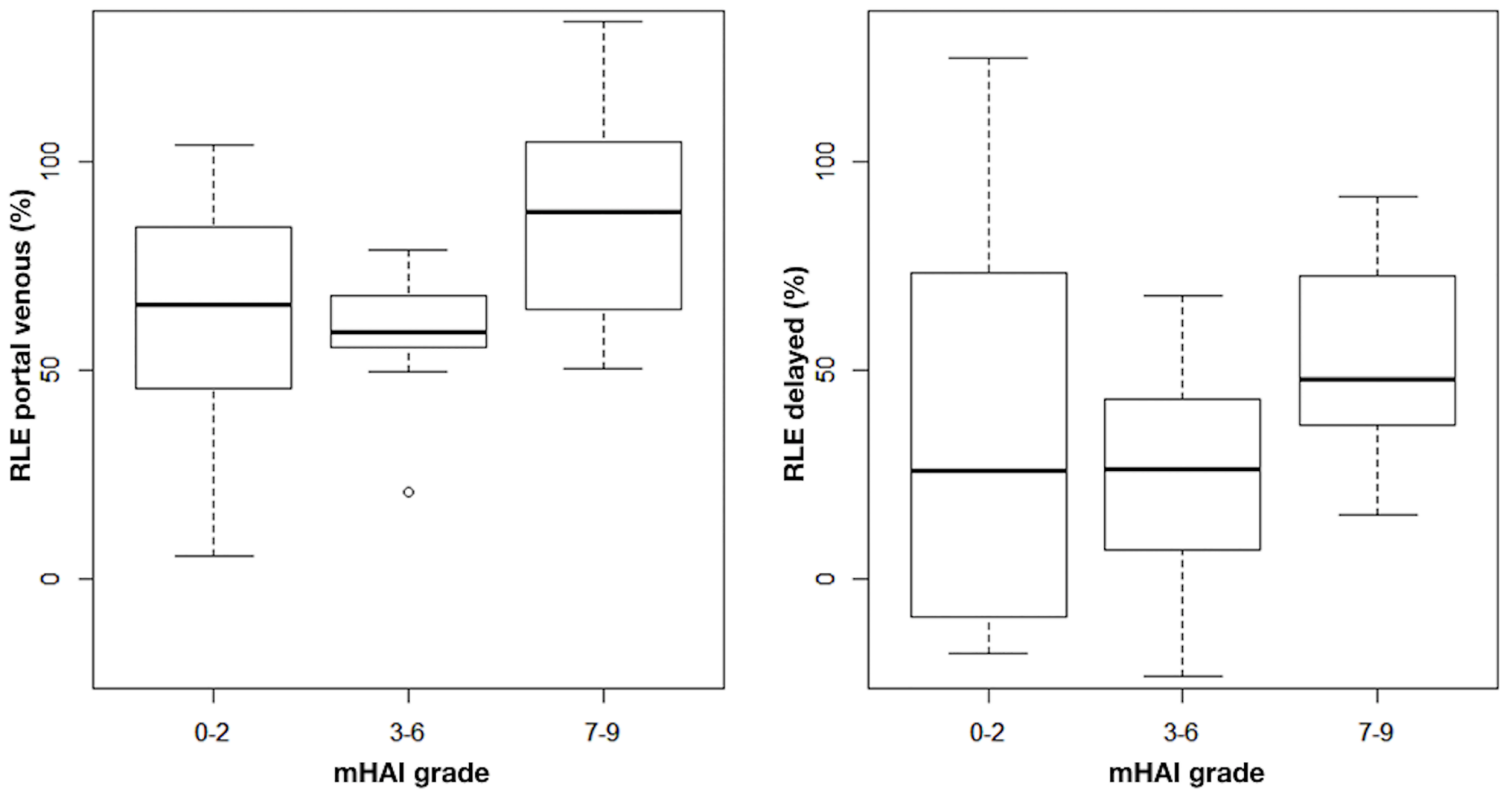
Portal venous (left) and delayed phase (right) relative liver enhancement (RLE) according to the Ishak modified hepatic activity index (mHAI).

Fig 5 depicts the RLE for activity of inflammation assessed by mHAI grade < 3, ≥ 3 and ≤ 6, and ≥ 7 points. Linear regression revealed a significant association of the portal venous phase RLE with mHAI grade ≥ 7 points [β = 25.5; 95% CI (2.53; 48.62); p = 0.04]. No association was observed for portal venous phase RLE with mHAI grade ≤ 6 (p ≥ 0.74) or mHAI and delayed phase RLE (p ≥ 0.45) (Table 3). Due to the small sample size with mHAI grade ≥ 7 points (RL N = 5; LL N = 3) (Table 2), further sensitivity analyses were not performed.

### Inter-rater agreement for quantification of relative liver enhancement

**Fig 6:**
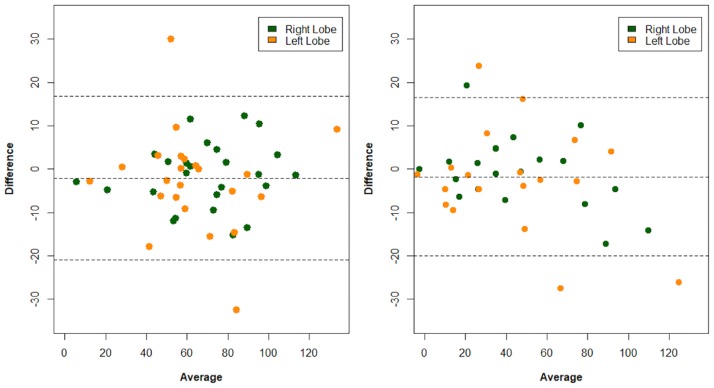
Bland-Altman plots of inter-rater agreement of the portal venous (left) and delayed phase (right) relative liver enhancement (RLE), including the right lobe and left lobe (y-axis: difference in RLE rating of two observers, x-axis: average of RLE rating of two observers). Median dashed line represents the bias, the upper and lower dashed line the 95% limits of agreement (LOA).

The quantification of RLE showed excellent inter-rater agreement both in the RL and LL. The inter-rater agreement of the portal venous phase was (Pearson correlation coefficient, 95% confidence interval): RL [r = 0.96 (0.10–0.98)], LL [r = 0.90 (0.77–0.96)]. The IRR for quantification of the RLE in the delayed phase was: RL [r = 0.98 (0.96–0.99)]; LL [r = 0.96 (0.90–0.98)], respectively. A Bland-Altman plot of both observers showed a bias of -2.05 [95% limits of agreement (-20.94 to 16.84)] and a bias of -1.79 [95% limits of agreement (-20.04 to 16.47)] for the portal venous and delayed RLE, respectively (Fig 6). For delayed phase RLE, the Bland-Altman plot depicts a wider distribution of data points in RLE measurements over 60%, while for lower RLE data points are closer to each other.

### T2 signal and CE alterations

Visual aspects of T2 hyperintense signal alterations on areas corresponding to the area of liver biopsy were found in 33 (RL: 19; LL: 14) of 66 histopathologically analyzed areas. A visible increase of contrast enhancement in delayed phase imaging was detected in 26 (RL: 14; LL: 12) of 66 areas (Table 2). In total, 7 out of 114 measurements were binary rated by the two operators and independently analyzed by the third reviewer. Binary ratings were predominantly found for T2 signal of the RL (N = 4) and LL (N = 2). Only one binary rating resulted in assessment of CE alterations (RL = 1; LL = 0). Images with binary ratings were reviewed by a third rater and decided positive in all cases. The IRR for detection of T2 hyperintense areas within the biopsy location was ‘substantial’ for the RL [Kappa = 0.77; 95% CI (0.548–0.981)] and ‘almost perfect’ for the LL [Kappa = 0.87; 95% CI (0.699–1.0)]. The IRR for detection of CE was ‘almost perfect’ for the RL [Kappa = 0.92; 95% CI (0.766–1.0)] and the LL [Kappa = 1.0; 95% CI (1.0–1.0)].

### Association of imaging parameters (CE, T2 signal) with histological inflammation and fibrosis

Using mixed-effects models to assess the association of visual aspects of T2 signal and CE, there was no statistically significant relationship between these imaging parameters and histopathological stages of fibrosis or mHAI grades (p ≥ 0.1 for all analyses). Details are listed in Table 3.

### Association of imaging parameters (CE, T2 signal, and RLE) and laboratory results

No association of laboratory parameters (Bilirubin, ALT, AST, γGT, AP, and IgG) and imaging parameters (T2, CE, and RLE) was found (S2 Table).

## Discussion

In PSC it is becoming increasingly recognized that non-invasive markers of liver inflammation and fibrosis are urgently needed for the assessment of disease progression and treatment response, particularly with respect to upcoming clinical trials [11]. This is the first study to analyze the association between T2 signal intensity, gadolinium-based contrast-enhanced multiphase MRI, and the histological severity of liver parenchymal inflammation and fibrosis in patients with PSC.

We propose that the relative liver enhancement in the delayed phase might serve as a valid marker for liver fibrosis in PSC and that it is superior to the visual assessment of T2 signal intensity and CE. According to the data in the present study a strong positive association between histological staging of fibrosis and delayed phase RLE can be observed, but not with portal venous phase RLE. This association is not observed for visual assessment of T2 signal intensity and CE. Peripheral wedge-shaped areas of T2 hyperintensity are frequently found in T2-weighted MRI of PSC livers. Until today no consensus exists whether inflammation or fibrosis contributes more to this finding. One study conducted on PSC patients found correlations of T2 signal intensity with histopathological signs of inflammation of hilar bile ducts, which causes obstruction of the venous and lymphatic drainage on biopsy examination [1]. However, the authors of this study also admitted that increased T2 signal is also observed in areas of confluent fibrosis or cirrhosis. The fact that T2 signal seems to be influenced by fibrosis and also by inflammation could be one more reason that findings obtained in this study lack a statistically significant association with stages of fibrosis or the modified hepatitis activity score.

A major strength of our study is the use of an extracellular gadolinium-based contrast agent. Correlations of a hepatocyte-specific (gadoxetic acid) contrast enhancement and liver fibrosis have been reported previously and describe a negative correlation of contrast enhancement and fibrosis stage (r = -0.79, p < 0.001) [48], (r = -0.545, p < 0.0001) [49]. However, the administration of a hepatocyte-specific contrast agent might additionally be useful in the setting of hepatocellular malignancy detection and evaluation of the biliary tree using T1 MRC [38], but at the disadvantage of increased material costs and acquisition times compared to conventional extracellular GBCA-enhanced MRI. These disadvantages of additional cost and time also apply for MRE, which has been shown to be able to distinguish between different stages of fibrosis and has therefore been proposed for evaluation of prognosis in patients with PSC [50].

In contrast to liver fibrosis, a weaker but significant association of portal venous RLE and mHAI grades ≥ 7 points was observed in this study cohort, whereas no associations of delayed RLE and mHAI grades were evident. The weak correlation of portal venous RLE and inflammation could possibly be influenced by hemodynamic changes of chronic liver disease (e.g. hepatic fibrosis or cholestasis in PSC patients) superimposed on the effects of portal venous phase MRI characteristics.

Our findings of delayed phase RLE are in line with the results of a previous multiphase GBCA-enhanced MRI study of patients with chronic and active hepatitis by Martin et al. [27]. In this study, arterial and delayed phase images were scored by three readers using feature categorization templates to quantify enhancement patterns. They found a high correlation of delayed phase liver enhancement scores with histopathological Scheuer stages of fibrosis (r = 0.96), but no association with histopathological Scheuer grades of inflammation (r = 0.071). However, enhancement patterns of the portal venous phase were not included in this study, and data obtained by the three readers were averaged despite only ‘fair’ agreement (Kappa 0.35 to 0.386). We believe, that the quantification of enhancement via RLE is more reliable than the quantification method applied by Martin et al. [27] with regard to IRR, which was considered ‘almost perfect’ on correlation analysis of this study. Visual quantification of T2 signal and CE reached slightly less favorable results.

To our knowledge, an association of extracellular GBCA portal venous phase enhancement and mHAI grades of liver inflammation have not yet been described. However, a recent study by Chen et al. [51] of N = 58 cases with chronic hepatitis found a significant association of gadoxetic acid DCE-MRI derived parameters arterial fraction (p = 0.033) and area under the curve (p = 0.047) with mHAI grades on liver histology [51]. Another study by Puustinen et al. [52] reported the feasibility of phosphorus-31 (^31^P) magnetic resonance spectroscopy for detection of inflammation in N = 12 autoimmune hepatitis patients. A significant positive correlation of phosphoenolpyruvate was observed with METAVIR grades of inflammation (r = 0.746; p = 0.005).

Recent studies published on PSC patients have mainly focused on liver function and not fibrosis. These studies evaluated T1 mapping and DCE-MRI. The reported T1 relaxation time decrease correlated with serum liver functional tests (AP, γGT, AST, ALT) as well as with the clinical scores Model for End-Stage Liver Disease (MELD) and PSC Mayo Risk Score [53]. Several other studies assessed the efficacy of different gadoxetic acid-enhanced MRI-based liver function indices in correlation with the indocyanine green (ICG) clearance (r = -0.354 to 0.574; p< 0.001) [54]. Another group evaluated the total and segmental liver function and volume by means of hepatocyte-specific DCE-MRI in a small cohort of N = 12 PSC patients. Authors reported significant correlations between biliary obstruction and segmental liver functional parameters (HEF Spearman´s rho -0.24; p < 0.05) and input relative blood flow (irBF Spearman´s rho -0.45; p < 0.05) [21].

Limitations of this study include the small number of PSC patients, especially those with histological Scheuer fibrosis staging of 3 or 4, and its single center set-up. In particular, the weakly significant correlation of portal venous RLE and higher mHAI scoring points must be interpreted with care, as this result was obtained with relatively small observations (mHAI ≥ 7; RL N = 5; LL N = 3). Before determining cut-off values for diagnostic purposes, larger follow-up multi center studies are needed to validate our preliminary observations. Additionally, liver biopsies were obtained up to 6 months before or after MRI scans, which could make exact relocation of the biopsy side difficult even with documentation in the laparoscopically performed procedure. An MRI-guided percutaneous biopsy or an even more precise upcoming technique of real-time MRI/Ultrasound (US) fusion-guided biopsy, which has already found application in prostate cancer would be of interest for future studies [55–57]. Lastly, two pathologists assessed liver histology by consensus, making it impossible to consider the effect of the IRR.

### Conclusion

The results of our study indicate the potential usefulness of extracellular GBCA enhancement, quantified by means of the RLE, for the staging of hepatic fibrosis and the identification of higher grades of hepatic inflammation in patients with PSC. Its easy application and use of existing equipment means this technique could be an interesting addition to the current imaging armamentarium of non-invasive fibrosis measurements and evaluation of disease activity, if validated in larger cohorts.

## Supporting information

S1 Table Intraindividual correlation of fibrosis stages and mHAI grades in patients with bilateral liver biopsy (N = 26). (DOCX)

S2 Table Laboratory parameters and their association with MRI parameters (T2, CE, RLE). (DOCX)

## Abbreviations

AUC: Area under the curve
CE: Contrast enhancement
CI: Confidence interval
DCE: Dynamic contrast-enhanced
GBCA: Gadolinium-based contrast agents
HEF: Hepatic extraction fraction
IRR: Inter-rater reliability
LL: Left liver lobe
LOA: Limits of agreement
MELD: Model for End-Stage Liver Disease
mHAI: Modified hepatic activity index
MRCP: Magnetic Resonance Cholangiopancreaticography
MRE: MR Elastography
MRI: Magnetic Resonance Imaging
MTT: Mean transit time
OR: Odds ratio
PSC: Primary sclerosing cholangitis
RL: Right liver lobe
RLE: Relative liver enhancement
ROC: Receiver operating characteristic
ROI: Region of interest
SI: Signal intensity
US: Ultrasound
