# Supplementary material for: Association of gadolinium-enhanced magnetic resonance imaging with hepatic fibrosis and inflammation in primary sclerosing cholangitis
Source: PLoS One. 2018 Mar 7;13(3):e0193929. doi: 10.1371/journal.pone.0193929 (PMC5841815; doi:10.1371/journal.pone.0193929)
Supplement: S1 Table — (DOCX) [file pone.0193929.s001.docx]

| N = 26 | Fibrosis LL | 0 | | 1 | | 2 | | 3 | | | 4 | |
| --- | --- | --- | --- | --- | --- | --- | --- | --- | --- | --- | --- | --- |
| Fibrosis RL | **0** | 3 | | 2 | | 1 | | 0 | | | 0 | |
|  | **1** | 0 | | 8 | | 3 | | 0 | | | 0 | |
|  | **2** | 1 | | 0 | | 2 | | 2 | | | 0 | |
|  | **3** | 0 | | 0 | | 1 | | 0 | | | 0 | |
|  | **4** | 0 | | 0 | | 0 | | 2 | | | 1 | |
| N = 26 | **mHAI LL** | **0** | **1** | **2** | **3** | **4** | **5** | | **6** | **7** | | **8** |
| mHAI RL | **0** | 1 | 1 | 0 | 0 | 0 | 0 | | 0 | 0 | | 0 |
|  | **1** | 0 | 4 | 5 | 0 | 1 | 0 | | 0 | 0 | | 0 |
|  | **2** | 0 | 1 | 1 | 2 | 0 | 0 | | 0 | 0 | | 0 |
|  | **3** | 0 | 0 | 0 | 0 | 0 | 0 | | 0 | 0 | | 0 |
|  | **4** | 0 | 2 | 0 | 1 | 0 | 0 | | 1 | 0 | | 0 |
|  | **5** | 0 | 0 | 0 | 0 | 0 | 0 | | 0 | 0 | | 0 |
|  | **6** | 0 | 0 | 0 | 0 | 0 | 1 | | 1 | 0 | | 0 |
|  | **7** | 0 | 0 | 0 | 0 | 0 | 0 | | 0 | 0 | | 1 |
|  | **8** | 0 | 0 | 0 | 0 | 1 | 0 | | 0 | 2 | | 0 |

**S1 Table** Intraindividual correlation of fibrosis stages and mHAI grades in patients with bilateral liver biopsy (N = 26).

Fibrosis: Spearman correlation coefficient r = 0.70; mHAI: Spearman correlation coefficient r = 0.71

Abbreviations: LL, left liver lobe; RL, right liver lobe; mHAI, modified hepatic activity index.
