# Supplementary material for: Association of gadolinium-enhanced magnetic resonance imaging with hepatic fibrosis and inflammation in primary sclerosing cholangitis
Source: PLoS One. 2018 Mar 7;13(3):e0193929. doi: 10.1371/journal.pone.0193929 (PMC5841815; doi:10.1371/journal.pone.0193929)
Supplement: S2 Table — (DOCX) [file pone.0193929.s002.docx]

**S2** **Table** Laboratory parameters and their association with MRI parameters (T2, CE, RLE).

| **Laboratory^1^** | **Count (N)** | **Mean ± SD** | **Contrast-enhancement^2^** | **T2 hyperintensity^2^** | **RLE pvp^3^** | **RLE dp^3^** |
| --- | --- | --- | --- | --- | --- | --- |
|  |  |  | **OR (p-value)** | | **Regression coefficient β**  **(p-value)** | |
| IgG (g/l) | 40 | 14.5 ± 4.5 | 2.436 (0.488) | 1.856 (0.686) | -4.646 (0.640) | 8.295 (0.580) |
| AP (U/l) | 39 | 249.6 ± 155.1 | 0.636 (0.689) | 1.185 (0.911) | -18.123 (0.069) | -0.413 (0.979) |
| Bilirubin (mg/dl) | 40 | 1.4 ± 1.7 | 3.222 (0.339) | 0.511 (0.690) | -5.379 (0.589) | 0.828 (0.956) |
| AST (U/l) | 40 | 89.7 ± 95.6 | 0.427 (0.487) | 2.468 (0.622) | -0.632 (0.953) | 17.581 (0.278) |
| ALT (U/l) | 40 | 134.8 ± 186.2 | 0.427 (0.487) | 0.977 (0.988) | -2.439 (0.819) | 24.946 (0.119) |
| γGT (U/l) | 40 | 345.7 ± 348.5 | 0.666 (0.767) | 1.491 (0.846) | -8.268 (0.522) | -3.243 (0.867) |

^1^ reference for all laboratory parameters is the normal laboratory parameter IgG, AP, etc.

^2^ based on mixed-effect logistic regression models with patient as random intercept, imaging (CE, T2) as dependent variables.

^3^ based on mixed-effects linear regression models with patient as random intercept, RLE (portal venous phase, delayed phase) as dependent variables.

Abbreviations: OR, Odds ratio; IgG, Immunglobulin G; AP, alkaline phosphatase; AST, aspartate-amino-transferase; ALT, alanine-amino-transferase; γGT, gamma-glutamyltransferase; RLE pvp, relative liver enhancement portal venous phase; RLE delayed, relative liver enhancement delayed phase.
